# Supplementary material for: Impact of Comorbidities of Patients with Psoriasis on Phototherapy Responses
Source: Int J Mol Sci. 2022 Aug 23;23(17):9508. doi: 10.3390/ijms23179508 (PMC9455671; doi:10.3390/ijms23179508)
Supplement: Supplementary file 1 [file ijms-23-09508-s001.zip › ijms-1842375-supplementary.pdf]

Table S1: Control and psoriatic patient groups used in this study.

		Control group	Psoriasis group
Gender	Men	49	101
	Women	51	99
	Total	100	200
Age	18-39 years	26	44
	40-59 years	39	111
	> 60 years	35	45
Phototherapy	UVA	No	85
	PUVA	No	36
	UVBBE	No	29
	UVA + UVBBE	No	50
Blood test		< 12 months	< 12 months
Informed consent		Yes	Yes

Table S2. Compounds used in this study.

Compound	Concentrations	Commercial branch	Catalog number	Target
WZB117	0.1 µM<br>1 µM<br>10 µM	Selleckchem	S7927	GLUT1
ATRA	1 µM	Sigma-Aldrich	R2625	Retinoic acid receptors (RARs)
Galactose	25 mM	Sigma-Aldrich	G5388	N/A
Glucose	25 mM	Sigma-Aldrich	G7021	N/A
NAD^+	1 mM	Sigma-Aldrich	N3014	N/A
